# Gestational Diabetes Is Associated With Abnormalities in Glucose Excursion in Early Pregnancy

**DOI:** 10.1155/ije/9922593

**Published:** 2025-12-30

**Authors:** Salwa Al-Maraghi, Safa Alzneika, Dhowa Al-Ali, Fatma Al-Haddad, Aly Mostafa Hassan, Sara Al-Thani, Noora Al-Khalaf, Farhan Cyprian, Mohamed Iheb Bougmiza, Bushra Nadeem, Hanan Khudadad, Stephen Beer, Mohammed Bashir, Abdul-Badi Abou-Samra, Suhail A. R. Doi

**Affiliations:** ^1^ Department of Population Medicine, College of Medicine, QU Health, Qatar University, Doha, Qatar, qu.edu.qa; ^2^ Department of Basic Medical Sciences, College of Medicine, QU Health, Qatar University, Doha, Qatar, qu.edu.qa; ^3^ Primary Health Care Corporation, Doha, Qatar, phcc.qa; ^4^ Department of Endocrinology, Hamad Medical Corporation Hospitals, Doha, Qatar; ^5^ Qatar Metabolic Institute, Hamad Medical Corporation, Doha, Qatar, hamad.qa

**Keywords:** dysglycemia, gestational diabetes, glucose excursion, glucose tolerance test, pregnancy, screening

## Abstract

**Aims:**

It is unknown if dysglycemia at 24–28 weeks of pregnancy is preceded by glycemic changes earlier in pregnancy. This study therefore examines the association between glucose excursion in the first 20 gestational weeks and the onset of gestational diabetes mellitus (GDM) in the early third trimester.

**Methods:**

A cohort study was conducted using data from the electronic medical record of the public health system in Qatar, and women with glycemic assessments done before 20 weeks and again in the early third trimester were assessed. The main outcome of the study was to examine glucose excursion (using Doi’s weighted average glucose; dwAG) in early pregnancy to see if it was indicative of GDM diagnosis at the usual time.

**Results:**

At the upper normal cutoff for dwAG (6 mmol/L), the sensitivity and specificity were 71.5% and 54.1%, respectively, and the diagnostic odds ratio was ∼3, meaning that for women beyond this threshold before 20 weeks gestation, they had, on average, a 3‐fold increase in odds of developing uGDM compared to women not meeting this threshold.

**Conclusions:**

It is concluded that early pregnancy glucose excursion remains in the normal range in women destined for third trimester GDM but is higher than that in those who do not develop GDM at this time and is a predictor of women at high risk early in pregnancy.

## 1. Introduction

Gestational diabetes mellitus (GDM) is a form of diabetes that arises during pregnancy, characterized by dysglycemia in women who did not have diabetes before pregnancy. It usually occurs in those women that come into pregnancy with some degree of beta cell dysfunction due to minor deficiencies in the β‐cell machinery [1] which may lead to decompensation later in pregnancy. Dysglycemia, considered sufficient to be designated GDM, usually happens in the early third trimester because this is when insulin resistance peaks due to placental factors such as increase in gluconeogenesis, oxidative stress, low‐grade chronic inflammation, and adipose tissue expansion. Eventually, 24–28 weeks of gestation has become established as the “usual” GDM (uGDM) screening window [2], and approximately 14.7% of women worldwide develop uGDM when assessed through the commonly utilized IADPSG criteria [3].

There has recently been an interest in examining the benefit of screening for “early” GDM (eGDM) in the period before 20 weeks of gestation. Screening for eGDM has mainly used either the usual adult prediabetes criteria or the same IADPSG criteria used for uGDM screening in the early third trimester. In women with first trimester prediabetes (HbA1c of 5.7%–6.4%) however, early therapy did not significantly lower the incidence of uGDM later in pregnancy [4]. An observational study [5] that investigated the pregnancy outcomes of eGDM versus uGDM, both diagnosed using the same IADPSG criteria [6], found that patients with eGDM were older with a higher likelihood of being obese, needing treatment and/or insulin therapy in comparison to patients with uGDM. The latter study also showed a significant difference when it came to some of the complications like cesarean delivery, preterm delivery, and neonatal intensive care (NICU) admission as they were higher within the eGDM group. However, more recent experimental studies have not confirmed these observational results and two key trials found no to minimal benefit for intervention in eGDM diagnosed using the IADPSG criteria [7–9].

While the debate around the early diagnosis of GDM continues, what has not been studied is if indeed the glycemic abnormalities in the usual period of testing (early third trimester) that are associated with uGDM do indeed evolve from the first trimester. Since the hallmark of the glycemic change associated with uGDM involves changes in both beta cell function and insulin resistance, these are reflected by glucose excursion measured through an oral glucose tolerance test (GTT). This study therefore examines the changes in glucose excursion in early pregnancy to determine if abnormalities in glucose excursion have already started earlier in those destined to develop uGDM. This study looks only at the evolution of glycemic changes over pregnancy and is not attempting to link these to maternal or fetal outcomes.

## 2. Subjects and Methods

### 2.1. Study Population, Design, and Setting

A cohort study design was employed using data obtained from CERNER through the Primary Health Care Corporation (PHCC) in Qatar. Consecutive records were screened for 35,069 pregnancies between December 2016 and March 2023 of women who delivered within the public health system in Qatar. Out of the pregnancies, women that had the following inclusion criteria were retained in the study:

a. Age > 17 years old.
b. Absence of preexisting diabetes mellitus types 1 or 2 or history of medications that may affect glucose metabolism.
c. Had at least two complete GTT assessments.
d. Had the first GTT measured at < 20 weeks and the second GTT between 22–30 weeks.
e. The time difference between the first and second GTT was > 6 weeks.

### 2.2. Oral GTT, Glucose Excursion, and GDM Diagnosis

GDM is usually diagnosed using the GTT with measurements taken at three time points (0, 60, and 120 min) after a 75‐g oral glucose load in the fasting state (≥ 8 h). The latter was done at two periods for each included participant—before 20 weeks of gestation and between 22–30 weeks of gestation (maintaining a minimum of 6 weeks between GTTs for women to be eligible). The test involves administering a 75‐g oral glucose load (a flavored solution containing 75 g dextrose) following an overnight fast of at least 8 h. Venous blood samples were collected at fasting, 1 h, and 2 h post‐glucose ingestion. In Qatar, the following preanalytical precautions are routinely in place: GTT samples are performed in the morning after fasting for at least 8 h. Patients do not smoke or consume anything, other than water, and do not engage in unnecessary physical activity throughout the duration of the GTT. Blood samples are collected into fluoride oxalate specimen tubes to inhibit glycolysis. The laboratory plasma glucose assay is enrolled and independently assessed in the College of American Pathologists proficiency testing program, and this follows the ADA 2011 recommendations for glucose measurement of ≤ 2.9% for imprecision and ≤ 2.2% for bias allowing a total analytical error (TAE) of ≤ 6.9%. This method of glucose estimation remained the same during the period of the study.

The hallmark of GDM is increased glucose excursion defined as abnormalities in the duration, frequency, and amplitude of glycemic variations around the mean blood glucose [10]. Therefore, glucose excursion before 20 weeks gestation was determined using a single measure called Doi’s weighted average glucose (dwAG). The latter is derived from the GTT and is a novel metric that concurs with area under the GTT but requires only three time points [11], representing the time points during the usual GTT performed in pregnancy (0, 60, and 120 min). The results of the three time points are then combined using the following expression: dwAG = (gtt0 × 0.28) + (gtt60 × 0.36) + (gtt120 × 0.36) and expressed as a single average glucose value in mmol/L.

GDM was diagnosed based on the IADPSG [6] criterion and is the most widely utilized GTT criterion for diagnosing GDM with thresholds derived from the results of the HAPO study [6]. With the IADPSG criteria, patients are binned into “GDM” or “No GDM” only and dysglycemia (of other magnitudes) cannot be diagnosed. GDM was diagnosed if any of the following plasma glucose thresholds are met or exceeded: fasting ≥ 5.1 mmol/L, 1 h ≥ 10.0 mmol/L, or 2 h ≥ 8.5 mmol/L.

### 2.3. Other Data Collected

Other data collected include the patient’s ethnicity that was classified based on regions into (1) Middle East and North Africa (MENA), (2) South Asia, (3) Western Europe and America, (4) Central and East Asia, and (5) Africa classified according to the UNICEF regional classification. In addition, data on maternal and fetal characteristics were collected including the mother’s age, height, and weight at the first antenatal visit and pregnancy weight gain. The BMI was calculated from height and weight and then classified into 3 groups: < 25 kg/m^2^ (normal), 25–30 kg/m^2^ (overweight), and > 30 kg/m^2^ (obese). We also collected data on the number of previous pregnancies for the mother while for the fetal characteristics, we only had access to the infant’s birth weight.

### 2.4. Management of Women in the Study

Women that met the IADPSG criteria at any time point (early or late) received management according to local guidelines that could have included medical nutrition therapy, metformin, or insulin as determined by the treating physician. Management was not recorded or analyzed in this study because in the eGDM group, the assessment preceded the management, while in the uGDM group, the women could have been under management at assessment but treatment (metformin or insulin) would be withheld for 24 h prior to the GTT if they were already on these medications. This study looks only at the evolution of glycemic changes over pregnancy and is not attempting to link these to maternal or fetal outcomes.

### 2.5. Statistical Methods and Data Analysis

Descriptive statistics were used to summarize the baseline characteristics of the study participants. The main outcome was the association between the development of GDM in the third trimester and continuous glucose excursion in early pregnancy measured through dwAG [11]. As glucose excursion was a continuous variable (dwAG), predictive performance of glucose excursion in early pregnancy was evaluated as a “test” for uGDM development using the IADPSG criteria at the usual time in pregnancy using diagnostic test methodologies. Linear regression was used to assess predictors of abnormal glucose excursion (dwAG) at < 20 weeks. These predictors were risk factors for development of GDM and included BMI, age, and ethnicity of the women at the first antenatal visit before 20 weeks gestation. These predictors were the only risk factors we had access to and since it is no longer considered best practice to select variables based on significance in univariate analysis, this was avoided. This was a predictive (not causal) model and therefore we do not need to follow a selection strategy as one would do for a causal analysis. We did not examine HbA1c because this was not the purpose of this paper. All statistical analyses were performed using Stata 17 (College Station, TX, USA), and exact *p* values were interpreted in terms of evidence against the hypothesized null effect model of data generation at our sample size [12].

## 3. Results

**Figure 1:**
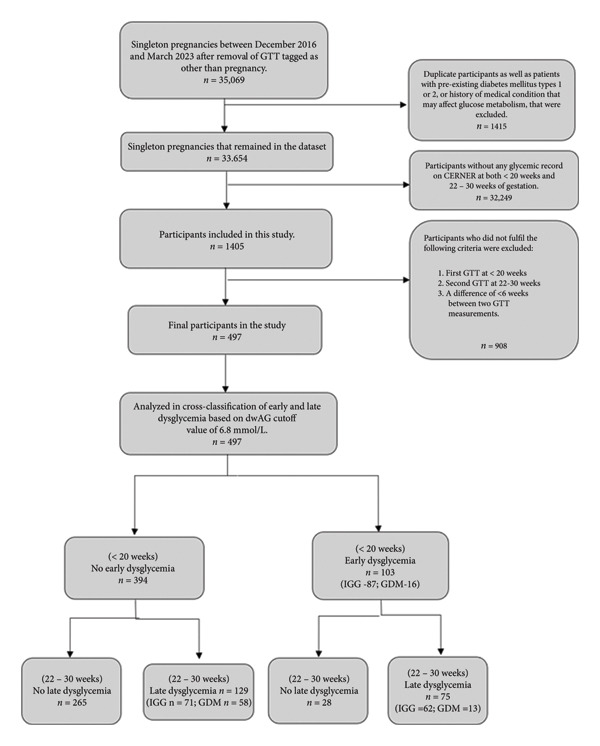
Flowchart of patient recruitment. Note that the cross‐classification in the last four boxes is based on the NPRP classification of hyperglycemia in pregnancy for information only. The main analysis in this paper, however, cross‐classifies GDM exclusively by the IADPSG criterion.

**Figure 2:**
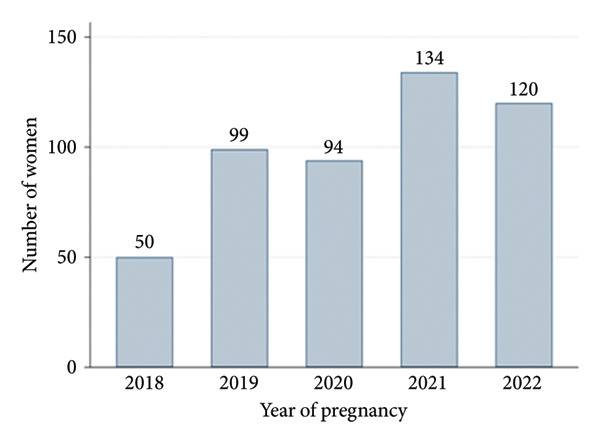
Distribution of included pregnancies by year depicting the rising trend of requesting an early GTT in this health system. Notably, none were requested in 2016/17 that met our inclusion criteria.

The selection of participants is depicted in Figure 1, and baseline characteristics of the study population are reported in Table 1. Out of the 35,069 pregnancies, 1405 pregnant women had at least two glycemic assessments (fasting plasma glucose [FPG], HbA1c, or GTT) over pregnancy, and after applying exclusions, a total of 497 women were eventually included in the study (Figure 1). The 908 women that were excluded from the study due to lack of the GTT (or not done at the right time) were compared to the 497 women included and they differed mainly on previous history of GDM which was much higher suggesting that this was used in decision making by clinicians for early screening with a GTT. There was an increasing trend, starting from 2018, of requesting an earlier GTT in this healthcare system (Figure 2). The cohort had an average age of 30.7 years, mean BMI of 27.8 kg/m^2^, mean maternal weight gain during pregnancy of 7.7 kg, and an average baby’s weight at birth of 3350.2 g. The median time (Table 1) of the early GTT was 15 weeks (IQR 12–17 weeks) and that for the late GTT was 25 weeks (IQR 24–27 weeks).

**Table 1:** Baseline characteristics of women excluded and included in the study.

Characteristics	Excluded participants^¶^	Included participants
	*N* = 908	*N* = 497
*Maternal and fetal characteristics*		
Maternal age (years)	28.2 (4.9)	30.7 (4.6)
BMI (kg/m^2^)	26.6 (5.3)	27.8 (5.1)
Early pregnancy weight (kg)	67.2 (14.8)	70.5 (13.7)
Late pregnancy weight (kg)	73.7 (14.6)	78.6 (14.3)
Height (cm)	158.9 (6.1)	159.4 (6.1)
Weight gain (kg)	6.2 (7.1)	7.7 (6.9)
No. of pregnancies	3.0 (2.0, 4.0)	3.0 (2.0, 4.0)
Baby’s weight at birth (g)	3202.7 (525.2)	3350.2 (458.0)
Previous history of GDM	14 (4.7%)	43 (22%)
*Glycemic parameters*		
Time of early GTT measures (weeks)	23.1 (20.1, 25.7)	15.2 (12.4, 16.6)
Time of late GTT measures (weeks)	25.8 (23.6, 28.6)	25.4 (24.0, 27.1)
NPRP GDM categories in early pregnancy		
NGG	NA	394 (79.3%)
IGG	NA	87 (17.5%)
GDM	NA	15 (3.0%)
hGDM	NA	1 (0.2%)
NPRP GDM categories in late pregnancy		
NGG	499 (64%)	293 (59%)
IGG	150 (19.3%)	109 (21.9%)
GDM	93 (12%)	75 (15.1%)
hGDM	37 (4.7%)	20 (4.0%)
Not on record	129	0
IADPSG GDM status in early pregnancy		
No GDM	GTT not done or done at the incorrect time	479 (96.4%)
GDM		18 (3.6%)
IADPSG GDM status in late pregnancy		
No GDM	612 (74.6)	353 (71.0%)
GDM	208 (25.4%)	144 (29.0%)
Not on record	88	0
FBG > 5.1 mmol/L in early pregnancy		
No GDM	801 (88.9)	484 (97.4%)
GDM	100 (11.1)	13 (2.6%)
Not on record	7	0
FBG > 5.1 mmol/L in late pregnancy		
No GDM	770 (88.4)	429 (86.3%)
GDM	101 (11.6)	68 (13.7%)
Not on record	37	0

*Note:* Mean (SD) or *N* (%) or median (Q1, Q3). NA: not available or done at an incorrect time.

^¶^To demonstrate the similarity between those excluded and included.

### 3.1. GDM and Dysglycemia Diagnoses—Early and Late Period

Using the IADPSG criteria, 3.6% met the criteria for GDM, and using the FPG alone, 2.6% met the criteria for GDM in the period before 20 weeks gestation. In the later period, between 22 and 30 weeks, GDM had increased 8‐fold (29%) and the increase was 5‐fold (13.7%) using the FPG alone (Table 2). Additionally, if there was GDM in the early period, half revert back to no GDM.

**Table 2:** Cross‐classification of uGDM (IADPSG criterion) by dwAG threshold in early pregnancy.

< 20 weeks	22–30 weeks		
	No GDM	GDM	Total
dwAG < 6 mmol/L	191 (54.1%, TN)	41 (28.5%, FN)	232
dwAG ≥ 6 mmol/L	162 (45.9%, FP)	103 (71.5%, TP)	265
Total	353	144	497

*Note:* Column percentages are shown. Note that 18/497 = 3.6% of the total cohort had GDM in the early period, and by the late period, 144/497 = 29.0% remained with GDM, an 8‐fold increase. Ten women (out of 18) with GDM in the early period reverted back to no‐GDM in the later period.

### 3.2. Diagnostic Performance of Early dwAG in Detecting GDM

**Figure 3:**
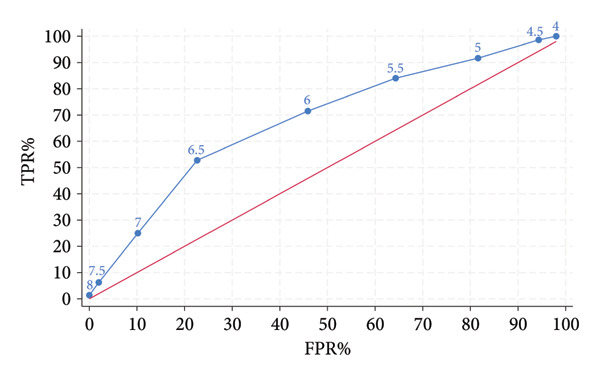
Receiver operating characteristic (ROC) curve for the early dwAG as a test of GDM at the usual time in pregnancy. *Note:* Numbers on the curve represent the dwAG threshold.

Figure 3 depicts the diagnostic performance of dwAG for predicting future diagnosis of GDM based on the IADPSG criteria. The area under the ROC curve for diagnosing GDM was 0.683 (95% CI 0.632–0.735). At a (dwAG) cutoff value of 6 mmol/L, the sensitivity and specificity were 71.5% and 54.1%, respectively. In addition, this cutoff had an odds ratio of ∼3, meaning that for women beyond this threshold in early pregnancy, they had, on average, a 3‐fold increase in odds of developing uGDM compared to women not meeting this threshold.

### 3.3. Factors Associated With Early dwAG Elevation

The results of linear regression analysis are depicted in Table 3 which assesses predictors of early dwAG. The coefficient for overweight women was 0.224 (95% CI, 0.023, 0.426; *p* = 0.029), whereas the coefficient for obese women was 0.380 (95% CI, 0.170, 0.590; *p* < 0.001). Both overweight and obese women therefore had higher dwAG values. Age was centered at 33 years old, and the coefficient of age was 0.027 (95% CI, 0.008, 0.045; *p* = 0.004). Age was also positively associated with dwAG values. Taking reference as MENA, the ethnic groups with higher dwAG values than the MENA group were South Asia, Western Europe and America, and Central and East Asia (Table 3), but for all three coefficients, there was weak to no evidence against the hypothesized null‐effect model of data generation at this sample size.

**Table 3:** Predictors of glucose excursion (dwAG) at < 20 weeks in a linear regression analysis.

Variable	Coefficient	95% CI	*p* value
BMI (kg/m^2^)			
< 25 kg/m2	Ref		
25–30 kg/m^2^	0.224	0.023, 0.426	0.029
> 30 kg/m^2^	0.380	0.170, 0.590	0.001
Age (years)	0.027	0.008, 0.045	0.004
Ethnic groups			
MENA	Ref		
Africa	−0.091	−0.394, 0.211	0.552
South Asia	0.149	−0.034, 0.332	0.110
Central and East Asia	0.142	−0.182, 0.467	0.389
Western Europe and America	0.423	−0.235, 1.082	0.207
Constant	5.794	5.61, 5.97	< 0.001

*Note:* Age was centered at 33 y.

## 4. Discussion

Previous studies have examined early dysglycemia using either measures of prediabetes or the GTT similar to what is done later in pregnancy [5]. These measures do not pick up early dysglycemia because they are not sensitive enough to changes occurring early in pregnancy [4]. This study demonstrates, for the first time, that abnormalities in glucose excursion seen with uGDM [13] have already started in early pregnancy (prior to 20 weeks gestation). However, there was no association between diagnosis of eGDM and uGDM by the IADPSG criteria because up to half of women diagnosed early may revert back to a non‐GDM level of hyperglycemia at 24–28 weeks in pregnancy. The latter has also been reported in other studies [9, 14] where a third of the women who had received a diagnosis of eGDM according to the IADPSG criteria did not have GDM on repeat GTT at 24–28 weeks’ gestation. The implication is that criteria for the diagnosis of GDM at the usual time do not apply early in pregnancy and the abnormality in glucose excursion we demonstrate in early pregnancy is not at a level that can be considered to be eGDM.

Reduced β‐cell mass, decreased β‐cell number, β‐cell dysfunction, or a combination of all three contributes to the ensuing hyperglycemia that defines GDM which underscores the inability of β‐cells to adequately respond to the increasing insulin resistance of pregnancy by releasing a sufficient amount of insulin [1]. In early pregnancy, the changes in glucose excursion are mainly driven by beta cell impairments coming into pregnancy while at 24–28 weeks, the glucose excursion may worsen for many reasons including worsening insulin resistance and desensitization of insulin receptors among other reasons. We have previously defined abnormal glucose excursion at 24–28 weeks gestation to be a dwAG > 6.8 mmol/L [13], and we find that only a fifth of women have this degree of dysglycemia coming into pregnancy that doubles to two‐fifths at 24–28 weeks gestation and only half of the latter develop GDM level dysglycemia (dwAG > 7.5 mmol/L [13]). This study finds that the abnormality seen in early pregnancy is even milder (dwAG > 6 mmol/L) and therefore below the threshold even for “dysglycemia” as we have defined it previously [11, 13]. The latter is a reflection of the fact that women have to have some degree of beta cell dysfunction coming into pregnancy to be at risk for GDM at 24–28 weeks gestation. Nevertheless, the level of dwAG in early pregnancy does not seem to correlate with the extent of failure of compensation by the beta cells and hence the reversion to normal of some women in later pregnancy. Perhaps this could partially have resulted from diet and nutritional interventions in those deemed (by usual criteria) to have eGDM; however, we did not have data on interventions to pursue this further.

The threshold for the dwAG at 6 mmol/L in early pregnancy had a sensitivity of 71.5% meaning that 71.5% of those with GDM at the usual time were above this threshold in early pregnancy. However, specificity was 54% and therefore half of women without GDM later in pregnancy also were above this threshold in early pregnancy. This underscores our previous observation that there may be women with higher dwAG values that retain the ability for compensation by beta cells later in pregnancy as insulin resistance increases. Perhaps the higher dwAG values in such women could be because they have more insulin resistance coming into pregnancy in relation to beta cell dysfunction as both affect glucose excursion as measured by the dwAG. Indeed, a study has reported that patients with eGDM had a higher likelihood of being obese when compared with those with uGDM [5]. Overall, dysglycemia that is destined to end up in uGDM seems to commence in early pregnancy as a milder abnormality in glucose excursion and the odds of developing uGDM increases 3‐folds in those with such mild early dysglycemia compared to those without. Additionally, the area under the curve of 0.74 (Figure 3) suggests that early pregnancy dysglycemia measured through the dwAG can moderately distinguish between those destined to develop uGDM and those not.

Several patient‐related factors were associated with higher early pregnancy dwAG levels in this study. BMI ≥ 25 kg/m^2^ was associated with higher early dwAG values consistent with what we suggested above. The dwAG for overweight women as well as for obese women suggested increases in dwAG in both groups over the normal group with strong evidence against the model hypothesis at our sample size. Older age also demonstrated increased dwAG values in early pregnancy with strong evidence against the model hypothesis at the current sample size. Compared to women from the MENA region, other ethnicities did suggest increases in dwAG (such as South Asia, Western Europe and America, or Central and East Asia) but with weak to no evidence against the model hypothesis at our sample size. These observations concur with the reports of previous observational studies [15, 16].

Our study does have certain limitations. First, because the study’s data came from the state of Qatar, there may be a limit to how broadly the results can be applied to other communities or nations, though there is expected to be generalizability as the dwAG has been validated in three continents [13]. Additionally, because the study uses retrospective data collection, there may be information bias. Stronger evidence on the development of dysglycemia and the effects of early screening on GDM‐related outcomes would come from additional prospective studies. When interpreting these results, these limitations should be taken into account.

In conclusion, this study presents a novel perspective that highlights the possibility of early biochemical changes in women destined to develop uGDM. This demonstrates that the evolution of glycemic changes starts early and women that do develop uGDM have already come into pregnancy with subtle dysglycemia that might not be seen with conventional glucose testing. Prediabetes levels of HbA1c or FPG have been used previously but it remains unclear if these are useful for diagnosis of eGDM or if it has any potential consequence for pregnancy [7–9]. Future studies can examine the utility of these findings to assist in early identification of women at risk for development of uGDM and if these subtle changes in early pregnancy can be a useful tool for risk stratification early in pregnancy. If so, this would be an opportunity for healthcare professionals to take a more proactive approach to GDM through early risk stratification.

**Nomenclature**
BMI: Body mass index
NGG: Normal gestational glycemia
IGG: Impaired gestational glycemia
GDM: Gestational diabetes mellitus
hGDM: High risk gestational diabetes mellitus
FPG: Fasting plasma glucose
dwAG: Doi’s weighted average glucose
UNICEF: United Nations Children’s Fund
ROC: Receiver operating characteristic
GTT: Oral glucose tolerance test 
NPRP: National Priorities Research Program
IADPSG: International Association of Diabetes in Pregnancy Study Groups
eGDM: Early (≤ 20 weeks of gestation) diagnosis of gestational diabetes mellitus
uGDM: Usual (24 to 28 weeks of gestation) diagnosis of gestational diabetes mellitus

## Ethics Statement

This study is an extension of a study on the association between gestational diabetes and newborn growth and developmental delays that was approved by the Primary Health Care Corporation in Qatar (PHCC/DCR/2022/12/070) and Qatar University Institutional Review Board (QU‐IRB 1990‐E/23). The approved deidentified data were used for this study.

## Consent

Retrospective deidentified health system data were utilized in this study, and therefore consent to participate was not required. All authors agree with the content and give consent for publication.

## Disclosure

The authors acknowledge that a poster was presented in the Eighth Qatar Diabetes, Endocrinology, and Metabolic Conference (QDEM‐8) 2024.

## Conflicts of Interest

The authors declare no conflicts of interest.

## Funding

This study was supported by the College of Medicine of Qatar University through the MSR project program.

## Data Availability

Data will be made available on reasonable request.

## Appendix

Stata analysis codes will be made available on request.
